# B cell regulation of the anti-tumor response and role in carcinogenesis

**DOI:** 10.1186/s40425-016-0145-x

**Published:** 2016-07-19

**Authors:** Marc Schwartz, Yu Zhang, Joseph D. Rosenblatt

**Affiliations:** Division of Hematology/Oncology, Department of Medicine, University of Miami Miller School of Medicine and Sylvester Comprehensive Cancer Center, 1120 NW 14th St., CRB 610, Miami, FL 33136 USA; Department of Medicine, University of Miami Miller School of Medicine, 1120 NW 14th St., CRB 610, Miami, FL 33136 USA; UM Sylvester Comprehensive Cancer Center, 1120 NW 14th St., CRB 610, Miami, FL 33136 USA

**Keywords:** B regulatory cells, Anti-tumor immunity, Carcinogenesis

## Abstract

The balance between immune effector cells such as T cells and natural killer cells, and immunosuppressive Treg cells, dendritic, myeloid and monocytic sub-populations in the tumor microenvironment acts to calibrate the immune response to malignant cells. Accumulating evidence is pointing to a role for B cells in modulating the immune response to both solid tumors and hematologic cancer. Evidence from murine autoimmune models has defined B regulatory cell (Breg) subsets that express cytokines such as IL-10, TGF-β, and/or express immune regulatory ligands such as PD-L1, which can suppress T cell and/or natural killer cell responses. Multiple murine tumor models exhibit decreased tumor growth in B cell deficient or B cell depleted mice. In several of these models, B cells inhibit T cell mediated tumor immunity and/or facilitate conversion of T cells to CD4^+^CD25^+^FoxP3^+^ T regs, which act to attenuate the innate and/or adaptive antitumor immune response.

Mechanisms of suppression include the acquisition of inhibitory ligand expression, and phosphorylation of Stat3, and induction of IL-10 and TGF-β, resulting in a Breg phenotype. Breg suppressive activity may affect diverse cell subtypes, including T effector cells, NK cells, myeloid derived suppressor cells (MDSC) and/or tumor associated macrophages. B cells may also directly promote tumorigenesis through recruitment of inflammatory cells, and upregulation of pro-angiogenic genes and pro-metastatic collagenases.

Breg infiltration has now been identified in a variety of solid tumor malignancies including but not limited to ovarian, gastric, non-small cell lung cancer, pancreatic, esophageal, head and neck, and hepatocellular carcinomas. Increasing evidence suggests that recruitment of B cells and acquisition of suppressive activity within the tumor bed may be an important mechanism through which B cells may modulate innate and/or adaptive anti-tumor immunity. B cell depletion in the clinic using anti-CD20 antibodies and/or inhibitors of BTK and/or other signaling pathways, may be a useful strategy for augmenting the anti-tumor immune response.

## Background

A subset of B cells identified as B-regulatory cells (Bregs) have recently emerged as major contributors to the pathogenesis of autoimmune and neoplastic disorders. In neoplastic disorders, Bregs are generated in response to signals from the tumor microenvironment and in turn promote tumor growth through interactions with T-regulatory cells (Tregs), myeloid-derived suppressor cells (MDSC), tumor-associated macrophages, CD4^+^ and CD8^+^ T lymphocytes, natural killer (NK) cells, and direct interactions with tumor cells. A role for B cells in anti-tumor immunity was first established by Brodt and Gordon [1] and Monach et al. [2], who demonstrated that tumor growth was diminished in B-cell depleted mice. Qin et al. [3] and Shah et al. [4] later demonstrated that B cells inhibit CTL-mediated tumor immunity. More recently Olkhanud et al. [5] and Tadmor et al. [6] showed that Bregs may facilitate the conversion of CD4^+^CD25^−^ T cells to CD4^+^CD25^+^FoxP3^+^ Tregs, which in turn attenuate the innate anti-tumor cellular immune response.

Effects of Breg cells in murine tumor models suggested that similar B regulatory activity would also be identified in human tumors. While some studies suggest that tumor-infiltrating B cells have a protective role against disease progression [7–12], other studies suggest that Bregs are upregulated in patients with solid tumor malignancies and are associated with enhanced tumor-aggressiveness and poorer prognosis [13–25]. In human lymphoid malignancies, malignant B cells appear to act as Bregs by suppressing the anti-tumor cellular immune response through expression of suppressive ligands [26–29]. This has led to successful targeting of B-regulatory functions of malignant B cells in lymphomas, which have thus far shown remarkably favorable outcomes in several clinical trials [26–28]. These results have lent credence to the notion that Breg activity may be modulating immune responses against non-lymphoid solid tumors. Bregs have now been identified in humans with a variety of solid tumor malignancies including ovarian, gastric, lung, colorectal, pancreatic, breast, esophageal, bladder, squamous cell, and hepatocellular carcinomas [13–22, 24, 25]. These observations suggest that targeting of Breg activity may be used to enhance immunotherapeutic outcomes.

### Breg subtypes in autoimmune disease and cancer

I.) Role of IL-10 in B cell regulatory function

IL-10 exerts anti-inflammatory effects in mouse and human cells via suppression of Th_1_ and Th_17_ responses, promotion of CD4^+^CD25^+^FoxP3^+^ Treg generation, and suppression of the release of macrophage- and monocyte-produced inflammatory cytokines [30–33]. B cells with regulatory function were initially described in murine models of autoimmune diseases such as experimental autoimmune encephalitis (EAE), collagen-induced arthritis (CIA), and inflammatory bowel disease (IBD), in which B cell deficient mice developed severe non-remitting disease that was ameliorated with B cell adoptive transfer [15–20, 34–39]. *Fillatreau* et al. [36] showed that mice which had recovered from EAE produced IL-10 in response to autoantigen, while mice incapable of producing IL-10 developed severe non-remitting EAE. Mice with IL-10 deficiency restricted to B cells also developed severe non-remitting EAE, which could be ameliorated through the adoptive transfer of IL-10-producing B cells from wild type (WT) mice that had recovered from EAE. CD40-CD40L interaction was recognized as an essential step in the generation of IL-10-producing B cells in response to autoantigen [36]. This and similar results in other mouse autoimmune models [32–34, 38–42] implicated IL-10 as a principal effector of B cell immune-regulatory activity. Decreased frequency and dysfunction of IL-10^+^ Bregs have been described in humans with various autoimmune disorders such as rheumatoid arthritis, systemic lupus erythematosus (SLE), inflammatory bowel disease, graft-versus-host disease, and vasculitides [43–52]. Enhancement of peripheral and organ-specific Bregs has been shown to be protective in patients with severe acute pancreatitis [53] but also has been associated with advanced histological fibrosis stages in patients with chronic hepatitis B virus infection [54], suggesting that Breg-mediated immune suppression may be beneficial in acute inflammatory states but harmful in chronic infection-mediated inflammatory states.

II.) Phenotypic markers of Bregs

In early mouse studies, IL-10 production was shown to be restricted mainly to a CD1d^hi^CD5^+^ (“B10”) subset that comprised roughly 1–3 % of splenic B cells [37, 38]. Other phenotypically distinct B cell subsets identified in humans exhibit immune regulatory properties through both IL-10 dependent and independent mechanisms. *Iwata* et al. [25] showed that IL-10-producing B cells in humans were predominantly found within a CD24^hi^CD27^+^ subset that was capable of suppressing monocyte cytokine production in vitro. *Blair* et al. [44] demonstrated that human CD19^+^CD24^hi^CD38^hi^ peripheral blood B cells suppressed CD4^+^ T cell IFN-γ and TNF-α production in vitro, with suppressive activity that was dependent on IL-10, CD80, and CD86. The latter two membrane proteins are key ligands for CTLA-4, a co-inhibitory immune checkpoint receptor expressed on activated effector T cells and Tregs [53, 55]. CD19^+^CD25^hi^ B cells have also been suggested to represent a Breg population in humans with the capability of suppressing CD4^+^ T cell proliferation and enhancing CTLA-4 and FoxP3 expression on Treg cells in vitro, in a manner dependent on TGF-β but not IL-10 [56]. CD5^+^ B cells have also been implicated in the suppression of anti-tumor immunity in humans through activation of Stat3 [57], a transcription factor that may be involved in production of IL-10 [58].

Additional B cell expressed surface antigens have been shown to confer regulatory properties. Programmed Death 1 Ligand (PD-L1) interacts with PD-1 on T cells to induce tolerance and limit effector T cell responses [59], and has recently shown to be expressed on human malignant B cells in several types of lymphoma including diffuse large B cell lymphoma (DLBCL), Hodgkin’s lymphoma, and follicular lymphoma [26–28]. Fas-Ligand (FasL), a member of the tumor necrosis factor pathway, interacts with its receptor FasR (CD95) to initiate a signaling cascade leading to apoptosis. B cell expressed Fas-L has been implicated in the induction of T_H_ cell apoptosis in HIV and EBV infections [60], and Fas-L expression on malignant B cells in lymphoid cancers such as chronic lymphocytic leukemia (CLL) may play a role in inhibiting anti-tumor responses by inducing T_H_ cell apoptosis [61]. *Lindner* et al. demonstrated that human Granzyme B-producing (GrB^+^) B cells are induced by IL-21-secreting T cells, which in turn function to suppress T cell proliferation via GrB-dependent degradation of the T cell receptor (TCR)- ζ-chain in vitro. GrB^+^ Bregs and adjacent IL-21-producing T cells were found in the microenvironment of several human tumors including breast, ovarian, cervical, colorectal, and prostate tumors [62]. These findings suggest that IL-21-dependent GrB^+^ Bregs may attenuate local anti-tumor immune responses in a manner similar to Tregs, by directly inhibiting the proliferation of CD4^+^ and CD8^+^ effector T cells.

### B regulatory cells suppress cellular immune responses and promote tumor growth in vivo through diverse mechanisms

I) Bregs promote tumor growth and metastasis through generation of Treg cells and suppression of effector T cell and NK cell responses

Several murine tumors including the MC38 colorectal cancer, EL4 thymoma, and EMT-6 mammary carcinoma models demonstrate reduced or absent growth in B-cell deficient mice (BCDM) compared to WT mice [4, 6, 63]. The anti-tumor effect conferred by B cell deficiency in all three models was associated with increased T cell and NK cell infiltration and more vigorous Th1 cytokine and cytolytic T cell responses, and in EMT-6 was also associated with decreased proliferation of CD4^+^FoxP3^+^ Tregs. Adoptive transfer of B cells from WT mice into tumor-bearing BCDM restored tumor growth and Treg proliferation and diminished CD8^+^IFN-γ^+^ and NK cell infiltration into the tumor bed. Interestingly, in contrast to several autoimmune models the adoptive transfer of either WT or IL-10^−/−^ B cells was capable of rescuing tumor growth in the EMT-6 model [63]. In the MC38 model, adoptive transfer of OX40L^−/−^ B cells was less efficient in rescuing tumor growth than WT B cell transfer, demonstrating a role for cognate interactions between OX40L and OX40 on B cells and T cells respectively in modulating the anti-tumor response [64].

Pulmonary metastasis of murine 4 T1 breast cancer requires inactivation of antitumor NK cells and expansion of Treg cells [5, 65]. *Olkhanud* et al. [5] identified a CD25^+^CD19^+^B220^+^ B cell subset designated as tumor-evoked Bregs (tBregs), that constitutively expressed Stat3 and was expanded in 4 T1 tumor-bearing mice. In vitro, mouse B cells treated with cancer cell-derived cultured media (CM) but not control CM was able to suppress T cell proliferation. Furthermore, T cell inhibitory activity was restricted to CD25^+^, but not CD25^−^ cancer-CM treated B cells. Interestingly, peripheral blood CD19^+^ B cells from healthy human donors treated with ovarian- and colon-cancer cell-derived CM upregulated CD25 and suppressed T cell proliferation in vitro as well, suggesting that human tumors may also induce a suppressive CD25^+^ Breg population.

Non-regulatory mouse CD4^+^CD25^−^FoxP3^−^ T cells developed increased expression of FoxP3 (thus demonstrating conversion to Tregs) when co-cultured with tBregs, but not control B cells, in a process that was dependent on TGF-β. WT mice also developed expansion of FoxP3+ Tregs in peripheral blood in vivo when injected with tBregs, but not control B cells. In T- and B-cell deficient NOD/SCID mice in which 4 T1 tumors grow at the primary site but do not metastasize, lung metastasis was restored with adoptive transfer of ex vivo converted Tregs (but not non-Tregs) or with the transfer of tBregs together with non-Tregs (but not tBregs alone), thereby demonstrating that lung metastasis of 4 T1 tumors was dependent on tBreg-mediated conversion of non-Tregs to Tregs in vivo. These results indicate that a unique CD25^+^CD19^+^B220^+^ B cell subset that constitutively expresses Stat3 may be expanded by tumors, and may in turn promote tumor metastasis through TGFβ-dependent conversion of non-Tregs to Tregs [5, 66].

II) Bregs impair antigen-specific anti-tumor vaccines through suppression of T and NK cell responses

Consistent with B regulatory cells having an immune-suppressive function, antigen-specific anti-tumor vaccines have been demonstrated to be more effective in the absence of B cells in several mouse tumor models. In the B16 melanoma mouse model, vaccination against either of the tumor-associated antigens (TAA) gp100 or TRP-2 using adenovirus-based vectors suppressed tumor growth in B-cell deficient, but not WT mice. The enhanced anti-tumor response against B16-associated tumor antigens in BCDM was dependent on the presence of NK cells [67]. In EL-4 thymoma implanted mice, *Oizumi* et al. [68] demonstrated using a highly immunogenic secreted form of gp96 heat shock protein as a vaccine, that effective tumor antigen-specific gp96-chaperone vaccination was achieved with a single vaccination in BCDM, while repeated vaccinations were required to achieve a similar level of immunity in WT mice. Increased CD8^+^ cytotoxic responses directed against EL-4-associated tumor antigens were observed in vaccinated BCDM compared to vaccinated WT mice. These results indicated that inhibition of effects mediated by immune-suppressive Breg cells may help maximize anti-tumor responses induced by vaccination.

III) Tumor-infiltrating B cells acquire LAP/TGFβ and PD-L1 expression and suppress T and NK cell responses

EMT-6 mammary tumors are rejected or show markedly diminished growth in BCDM but growth is restored in BCDM reconstituted with B cells [63]. Using the EMT-6 model *Zhang* et al. [69] demonstrated that tumor-infiltrating B cells (TIL-B) develop increased expression of LAP/TGF-β, CD80, CD86, and PD-L1 in vivo compared to splenic B cells. Development of a similar B cell immunosuppressive phenotype also occurred when B cells were co-cultured in vitro with EMT-6 cells, and was dependent on direct physical B cell: tumor cell contact. Functionally, these TIL-B cells demonstrated greater ability to suppress CD4^+^ and CD8^+^ T cell proliferation in response to anti-CD3/anti-CD28 co-stimulation, and also markedly suppressed NK cell proliferation in response to IL-15 compared to splenic B cells. Monoclonal antibodies directed against TGF-β or PD-L1 dramatically suppressed EMT-6 tumor growth in WT mice, suggesting a potential therapeutic strategy for targeting this specific B cell subpopulation. In addition, TIL-B but not splenic B cells were capable of secreting IL-10 following stimulation, suggesting that an IL-10 secreting subpopulation was predominantly contained within the TIL-B population. These results point to the local acquisition of immunosuppressive properties by B cells migrating into the tumor bed through intimate contact with the tumor cells. Mechanism(s) underlying this functional transition and migration, as well as evidence for similar activity in human tumors are being actively investigated.

IV) T2-MZP B cells accumulate in tumor-draining lymph nodes and promote metastasis independently of IL-10

B16-F10 melanoma grows significantly more slowly, but is not completely rejected in BCDM relative to WT mice [4]. Marked B cell accumulation in tumor-draining lymph nodes (TDLN) of mice implanted with B16-F10 melanoma tumors precedes the development of metastases. *Ganti* et al. [70] showed that T2-MZP (B220^+^CD23^+^IgM^hi^CD21^hi^) B cells preferentially accumulated in the TDLN of tumor-bearing mice. Both tumor-implanted BCDM and WT mice demonstrated accelerated tumor growth when reconstituted with T2-MZP B cells, but not other B cell subtypes. Frequencies of IL-10-secreting B cells and FoxP3^+^ Tregs were not affected by T2-MZP B cell transfer. In this model, B16-F10 melanoma induces accumulation of T2-MZP Bregs in TDLN, which in turn promote tumor growth and metastasis, and appear to do so independently of IL-10 and/or Tregs. Further investigations are warranted to determine the mechanism of Breg activity in this unique B cell subset.

V) Bregs promote tumor growth and metastasis through “education” of myeloid-derived suppressor cells

MDSCs are key regulators of tumor growth and metastasis in the tumor microenvironment [71]. Using the B16 melanoma model in which tumor growth is inhibited in BCDM but restored with adoptive B cell transfer, *Bodogai* et al. [72] showed that adoptive transfer of MDSCs from B16-implanted *WT* mice into tumor-bearing BCDM restored tumor growth to the same degree as did adoptive transfer of B cells from B16-implanted WT mice. Conversely, adoptive transfer of MDSCs from B16-implanted *BCDM* into tumor-bearing BCDM did not restore metastasis.

In human cell lines, myeloid cells sort-purified from healthy donor PBMCs depleted of T and NK cells and treated with CM from MDA-MB-231 breast cancer cells were able to suppress T-cell proliferation in vitro. However, myeloid cells from donor PBMCs depleted of T, NK, *and B cells* and treated with cancer-derived CM failed to suppress T cell proliferation.

These results indicate that the immune-suppressive and tumor-promoting functions of MDSCs may be acquired through “education” by Bregs. The nature of B cell mediated education of MDSC is unclear, but clearly indicates a role for Breg in modulating myeloid suppressor cell activity, in addition to aforementioned effects on T and NK cells, thereby shaping the overall immune microenvironment.

VI) The role of Stat3 activated Bregs in promoting tumor growth and metastasis via generation of Treg cells and promotion of angiogenesis

Consistent with the finding that CD19^+^CD25^+^ tumor-evoked Bregs (tBregs) constitutively express Stat3 and promote tumor growth and metastasis by TGFβ-dependent conversion of non-Tregs to Tregs [5], *Lee-Chang* et al. [73] demonstrated that treatment with non-cytotoxic doses of resveratrol (RSV), a potent inhibitor of Stat3 phosphorylation, suppressed proliferation of tBregs and FoxP3^+^ Tregs in vitro, and inhibited growth of B16 and 4 T1 murine tumors in vivo. In in vitro experiments using naïve B cells treated with 4 T1 cell-derived CM, low-dose RSV blocked tBreg generation, Treg generation, and reversed tBreg/Treg-mediated suppression of T cell proliferation. tBregs treated with RSV also had decreased expression of phosphorylated Stat3 (pStat3) and TGFβ compared to mock-treated tBregs. Furthermore, RSV-treated tBregs adoptively transferred into 4 T1 tumor-bearing mice lacked the ability to expand FoxP3^+^ Tregs in vivo and promote lung metastasis as compared to mock-treated tBregs. These findings suggest that inhibition of Stat3 phosphorylation in Bregs, by RSV or other methods, may inhibit tumor growth by preventing the downstream local elaboration of TGF-β and subsequent promotion of FoxP3^+^ Tregs.

JSI-124 (cucurbitacin I), a potent Stat3 inhibitor, also inhibited growth of 4 T1 murine tumors. 4 T1-implanted mice treated with JSI-124 suppressed tumor growth compared to non-treated mice, and B cells isolated from treated mice had decreased expression of Stat3. Consistent with the hypothesis that inhibition of B cell-expressed Stat3 was responsible for the anti-tumor effect of JSI-124, Stat3^low^ B cells from treated mice exhibited a *tumor-suppressive* effect when injected into 4 T1-bearing mice, while injection of Stat3^high^ B cells from non-treated mice *promoted* tumor growth [74].

*Yang* et al. [75] showed that increased tumor growth mediated by Stat3 expression in B cells was associated with increased tumor angiogenesis. In T- and B-cell deficient Rag1^−/−^ mice, growth of B16 and Lewis Lung cancer (LLC) tumors was augmented following the adoptive transfer of Stat3^+/+^ B cells, while growth was diminished with adoptive transfer of Stat3^−/−^ B cells. Augmented tumor growth with Stat3^+/+^ B cell adoptive transfer was associated with increased tumor angiogenesis in vivo by Matrigel assay and in vitro in B cell: endothelial cell co-culture assays, and increased expression of pro-angiogenic genes by RNA in vivo. TIL-B cells with persistently-activated Stat3 has also been identified in several human tumors including melanoma, gastric, lung, liver, and prostate cancers [75]. Furthermore, Stat3 activity in human tumor tissues was associated with increased density of TIL-B cells and significantly increased intratumoral angiogenesis.

*Zhang* et al. [57] recently showed that CD5 positivity among CD19 cells strongly correlated with levels of Stat3 expression in human lung and prostate tumor tissues and in corresponding TDLN, indicating that Stat3-expressing Bregs may be contained within a CD5^+^ B cell population in human tumors.

Collectively these studies indicate an important role for Stat3 in conferring an immunosuppressive phenotype to Breg cells, mediated in part through local elaboration of TGF-β and through the induction of pro-angiogenic gene expression. Inhibition of Stat3 signaling may therefore be a useful means of reducing or reversing Breg promotion of tumor growth.

VII) CD19^±^CD1d^high^CD5^±^ Bregs infiltrate pancreatic neoplasms and mediate tumor growth via IL-35 signaling

*Pylayeva-Gupta* et al. [76] recently showed that prominent B cell infiltrates are frequently present in human pancreatic intraepithelial neoplasia (PanIN) and in pancreata of mice harboring Kras-driven pancreatic neoplasms. In addition, implantation of pancreatic ductal epithelial cells expressing oncogenic Kras (Kras^G12D^) into WT mice pancreata leads to infiltration of B cells adjacent to the newly developing tumor. Kras^G12D^ pancreatic tumor growth is diminished in BCDM but tumor growth is restored following B cell adoptive transfer, which is accompanied by de novo tumor infiltration with the adoptively transferred B cells.

Since the CD1d^hi^CD5^+^ B cell population has been shown to mediate immune-suppression via IL-10 secretion [37, 38] and IL-35 [77] in mouse autoimmune and tumor models, *Pylayeva-Gupta* et al. investigators hypothesized that CD1d^hi^CD5^+^ B cells would also regulate immune-suppression and promote tumor growth in the mouse pancreatic ductal adenocarcinoma (PDAC) model. Adoptive transfer of CD19^+^CD1d^hi^CD5^+^ B cells but not CD19^+^CD1d^lo^CD5^−^ B cells rescued tumor growth in BCDM inoculated with Kras^G12D^. Adoptive transfer of IL-10^−/−^ B cells was capable of rescuing tumor growth in BCDM inoculated with Kras^G12D^ to the same extent as WT B cells, however tumor growth remained suppressed with adoptive transfer of IL-12α^−/−^ B cells, indicating that promotion of tumor growth by infiltrating CD1d^hi^CD5^+^ B cells in this PDAC model is mediated by IL-35, a heterodimer comprised of p35 and EBI3 subunits encoded by the genes IL12α and EBI3, respectively.

IL-35^+^ Bregs have been shown to inhibit Th_1_ and Th_17_ cells and promote Treg expansion in a mouse model of autoimmune uveitis [77]. IL-35 has also been shown to promote growth of human pancreatic cancer cells in vitro [78], and has been shown to be upregulated in the sera of patients with pancreatic cancer [79]. Together, these results suggest that B cells may infiltrate pancreatic neoplasms and promote tumor growth via suppression of antitumor immunity through IL-35 secretion. IL-35 mediated Breg suppression of tumor immunity may also be a suitable target for therapeutic intervention in select human tumors.

VIII) Bruton’s tyrosine kinase (BTK) expression in B cells and macrophages, as a target for regulation of immune-suppressive phenotypes

Previous findings by Affara et al. [80] and Andreu et al. [81] suggested that TIL-B cells promote tumor growth in squamous cell carcinoma (SCC) models through interactions with Ig receptor FcRγ + myeloid cells and subsequent repolarization of TAM towards an immune-suppressive type (“M2”-type). *Gunderson* et al. [82] showed that mouse PDAC tumors were heavily infiltrated with B cells and FcRγ + myeloid cells, and furthermore that tumor growth was diminished in BCDM and Ig-receptor null FcRγ^−/−^ mice. Based on these observations, investigators hypothesized that signaling pathways common to both B cells and macrophages, such as BTK signaling, may be involved in the suppression of antitumor immunity by infiltrating lymphocytes in PDAC tumors. In accordance with this hypothesis, activated BTK (pBTK) was identified in murine PDAC tumors in single-cell suspensions and was most prominent in CD19^+^ B cells and CD11b^+^ myeloid cells.

In in vitro assays using PDAC-derived B cells co-cultured with macrophages, PDAC-derived B cells enhanced macrophage expression of Th2 cytokines. In contrast, the pre-treatment of PDAC-derived B cells with the BTK inhibitor ibrutinib instead enhanced macrophage expression of Th1 cytokines. These results indicate that inhibition of BTK in tumor-infiltrating B cells may promote macrophage repolarization from an immune-suppressive, tumorigenic M2-type toward a pro-inflammatory, anti-tumor M1-type.

In vivo, early stage PDAC tumor-bearing mice treated with ibrutinib exhibited significantly diminished tumor growth. Treatment of late-stage tumor-bearing mice treated with ibrutinib plus gemcitabine resulted in significantly diminished tumor growth compared to gemcitabine alone. BTK may therefore represent a novel potential target for reduction of Breg cell activity in cancer, and furthermore BTK inhibition may augment the efficacy of chemotherapy.

Ibrutinib has recently been approved by the FDA for treatment of patients with refractory or p53-mutated CLL [83], as well as patients with relapsed mantle cell lymphoma [84]. These findings have provided a rationale for clinical trials examining the use of ibrutinib as adjunctive therapy to gemcitabine and nab-paclitaxel in metastatic pancreatic carcinoma (NCT02562898, NCT02436668).

IX) A link between 4-1BBL expression on B cells and the generation of cytotoxic Granzyme-B^+^ T cells

Interestingly, depletion of B cells with an anti-CD20 antibody (α-CD20 mAb) in WT mice prior to inoculation with 4 T1 cells blocked metastasis of 4 T1 tumor, consistent with findings observed in genetically B-cell deficient mice [85]. However, administration of α-CD20 mAb to WT mice with established 4 T1 tumors unexpectedly resulted in significantly enhanced tumor growth and metastasis.

*Bodogai* et al. [85] reconciled this paradoxical result by demonstrating that α-CD20 mAb treatment of 4 T1 tumor-bearing mice enriches for B cells that are phenotypically CD20^low^ and have enhanced T cell-suppressive effects ex vivo compared to B cells from control IgG2a-treated tumor-bearing mice. Consistent with these findings in mice, co-culture of human peripheral blood B cells with human breast cancer or colon cancer cells resulted in emergence of a CD20^low^ Breg population that suppressed T-cell activity and was sustained after treatment with α-CD20 mAb in vitro.

Screening for TLR ligands capable of inactivating Bregs revealed that the TLR9 ligand CpG-ODN was able to upregulate CD20 and block the T cell-suppressive effects of human and murine tBregs in vitro*.* Treatment of 4 T1 tumor-bearing mice with CpG-ODN abrogated lung metastasis, and ex vivo B cells derived from CpG-ODN treated tumor-bearing mice induced T cell proliferation and expansion of GrB^+^CD8^+^ cytolytic T cells. Enhancement of metastasis by α-CD20 mAb treatment was completely reversed in vivo when α-CD20 mAb-treated tumor-bearing mice were adoptively transferred with CpG-ODN-pretreated B cells from syngeneic naïve mice. In contrast, adoptive transfer of mock-treated B cells minimally reduced the enhanced lung metastasis following α-CD20 mAb treatment.

4-1BBL (CD137L) is an immune co-stimulatory receptor and a member of the TNF receptor family, which activates CD8^+^ T cells and NK cells via cross-linking of 4-1BB (CD137) expressed primarily on T cells [86]. *Bodogai* et al. [85] investigators observed that human and murine CD20^low^ Bregs also had reduced 4-1BBL expression compared to normal B cells. Treatment with CpG-ODN reversed the reduced expression of 4-1BBL on murine and human Bregs in vitro as well as in vivo in tumor-bearing mice, resulting in emergence of a CD20^high^4-1BBL^high^ B cell subset.

Consistent with the results seen in the 4 T1 murine model, the depletion of B cells using α-CD20 mAb in mice inoculated with EMT-6 did not result in tumor rejection as expected, and was associated with a paradoxical increase of CD4^+^FoxP3^+^ Tregs in the spleens of treated mice, although a role for 4-1BBL was not investigated in the EMT-6 model [63].

These results suggest that tumors can induce a population of CD20^low^4-1BBL^low^ immune-suppressive Bregs that are relatively enriched following α-CD20 mAb treatment. Relative enrichment of CD20^low^4-1BBL^low^ Bregs therefore is a plausible explanation for the failure of rituximab to provide a clinical benefit when combined with IL-2 immunotherapy for patients with renal cell carcinoma and melanoma [87]. Treatment strategies directed toward facilitating the conversion of 4-1BBL^low^ Bregs to immune-stimulatory 4-1BBL^high^ B cells, as demonstrated using CpG-ODN in the 4 T1 model [85], may therefore be a useful strategy to reverse tumor-mediated induction of suppressive Bregs.

X) IgA^+^CD138^+^PD-L1^+^IL-10^+^ Bregs impede expansion of anti-tumor cytotoxic T-lymphocytes (CTL) induced by oxaliplatin chemotherapy

In both the TRAMP and Myc-Cap (MC) mouse models of metastatic prostate carcinoma (PC), large tumors (>0.7 g, >350–400 mm^3^ respectively) were resistant to treatment with low-dose oxaliplatin in WT mice but were sensitive to treatment in BCDM [88]. The anti-tumor effect conferred by B cell deficiency was associated with enhanced CD8+ cell infiltration in both tumor types. Treatment of tumor-bearing WT mice with oxaliplatin was associated with emergence of a tumor-infiltrating CD19^+^CD138^+^IgA^+^ plasma cell population that expressed IL-10, PD-L1, phosphorylated Stat3, and Fas-L. Reconstitution of tumor-bearing BCDM with WT B cells, but not PD-L1^−/−^ or IL-10^−/−^ B cells, could restore tumor growth in oxaliplatin-treated mice, implicating both PD-L1 and IL-10 in mediating the Breg suppressive effects. In addition, ablation of B cell TGFβR2 inhibited oxaliplatin-induced IgA^+^ plasma cell generation, blocked induction of tumoral PD-L1^+^ by oxaliplatin and was associated with increased tumoral CTL density and IFN-γ production, thus implicating TGF-β signaling in the generation of this Breg subpopulation. Large MC tumors resistant to treatment with oxaliplatin monotherapy showed diminished tumor growth when treated with oxaliplatin plus anti-PD-L1, but not with anti-PD-L1 alone.

In human prostatectomy samples, CD8^+^ and CD20^+^ cell density was higher in patients with PC compared to healthy controls, and correlated significantly with advanced stage and treatment failure [88]. IL-10-producing IgA^+^CD138^+^ plasma cells were also present in human PC samples but were more abundant in metastatic PC and therapy-resistant PC compared to early stage disease.

These results indicate that treatment with an immunogenic chemotherapeutic agent, oxaliplatin, may induce a CD138^+^IgA^+^PD-L1^+^IL-10^+^ Breg population generated via a TGFβ-dependent pathway that limits the effectiveness of oxaliplatin by inhibiting intratumoral CTL infiltration. This further suggests that the successful treatment of large prostate tumors with oxaliplatin may require the elimination of these immunosuppressive IgA^+^ plasma cells that are present in both mouse and human PC, and suggests a rational approach to augmenting beneficial chemotherapy effects on the anti-tumor immune response. Targeting of the PD-L1/PD-1 axis may be an especially useful strategy in this regard.

**Fig. 1:**
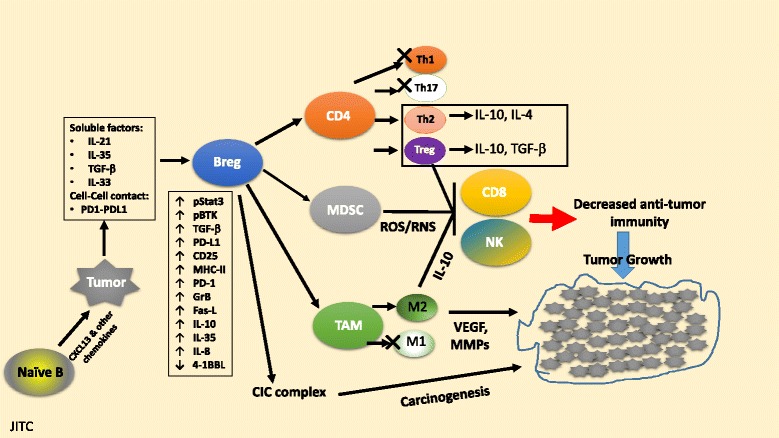
Tumor educated B regulatory cells suppress anti-tumor immunity. Tumor cell secreted chemokines such as CXCL13, may attract naïve B cells into the tumor microenvironment. Tumor cells and tumor infiltrating Treg cells may express inhibitory molecules (e.g. PD-L1) and/or secrete cytokines (e.g. IL-21, IL-35, or TGF-β) that may promote differentiation of B cells leading to development of a B regulatory phenotype (Breg cells). Breg cells may undergo activation of Stat3, and also upregulate key regulatory or inhibitory molecules such as PD-L1, CD25, CD86, LAP/TGF-β, and Granzyme B, and secrete cytokines, such as IL-10, IL-35 and TGF-β. Breg cells can suppress T and NK cell activation, proliferation and function in vivo and also ‘educate’ MDSC and tumor associated macrophages (TAM) to suppress anti-tumor immunity. Breg cells have also been noted to support natural Treg cell expansion and the conversion of effector CD4+ T cells into inducible Treg cells. Breg cells may also facilitate macrophage differentiation into TAM-M2 macrophages and increase local inflammation and thereby promote carcinogenesis in certain settings. Further details and supporting references in text

A summary of reported B reg phenotypic markers and functional attributes is presented in Table 1, and several of the key reported interactions reviewed above are schematically represented in Fig. 1.

**Table 1:** Breg markers of immune suppression and tumorigenesis

Breg marker	Mechanisms	Phenotype(s)	Cancer type(s)	References
IL-10	↓Th1/Th17; ↑Treg generation	Human:	HNSCC, lung, esophageal, ovarian, glioma, gastric	[5, 13–22, 25, 37, 38, 44, 56, 65]
		CD19^+^CD24^hi^CD38^hi^		
		CD19^+^CD1d^hi^CD5^+^		
		CD19^+^CD24^hi^CD27^+^		
		CD19^+^CD25^hi^		
		Mouse:		
		CD19^+^CD1d^hi^CD5^+^		
		CD19 + CD25 + B220+		
Granzyme-B	↓T cell proliferation via degradation of TCR-zeta chain	Human:	Breast, ovarian, cervical, colorectal, prostate	[62]
		CD19 + CD38 + CD1d + IgM + CD147+		
IL-35	↓Th1/Th17; ↑Treg generation	Human:	Pancreatic	[76–79]
		CD19 + CD35+		
		Mouse:		
		CD19^+^CD1d^hi^CD5^+^		
TGFβ	↑Treg generation	Human:	Multiple tumor types	[5, 20, 44, 65]
		CD19^+^CD24^hi^CD38^hi^		
		Mouse:		
		CD19^+^CD25^+^B220^+^		
Stat3	↑Treg generation; ↑angiogenesis	Human:	Melanoma, gastric, lung, liver, prostate	[57, 73–75]
		CD5+		
		Mouse:		
	↑IL-10, ↑TGFβ	CD19^+^CD25^+^B220^+^		
Lymphotoxin-α/β	Activation of IKKα and STAT3	Mouse:	Castration-resistant prostate (mice)	[93]
		CD19 + LT+		
PD-L1	Promote T cell anergy via interaction with PD1	Human:	B cell lymphomas, prostate	[26–28, 69, 88]
		Malignant B cells		
		IgA + CD138+		
		Mouse:		
		IgA + CD138+		
PD-1	Promote T cell anergy	Human:	Hepatocellular	[15]
		CD19 + CD5^hi^CD24^−/+^CD27^hi/+^CD38^dim^		
TNFα	↑IL-10+ Bregs, ↓CD8 + IFNγ Tcells	Mouse:	Squamous cell skin CA (mice)	[90]
		CD19 + TNFα+		
OX40L	↑Th2 skewing, ↓CD8 + IFNγ Tcells	Mouse:	Colorectal (mice)	[64]
		CD19 + OX40L+		
CD80, CD86	↓T cell proliferation via interaction with CTLA-4; may also ↑T cell proliferation via interaction with CD28	Human	Multiple tumor types	[30, 44, 69]
		CD19^+^CD24^hi^CD38^hi^		
IL-8	Upregulation of androgen receptor and downstream MMPs	Human:	Bladder	[22]
		CD19 + IL-8+		
FasL	Induce T cell apoptosis via binding to Fas	Human:	CLL	[60, 61]
		Malignant B cells		
CD40L	Interact with CD40 on malignant cells to stimulate ↑IL-10, ↑TGFβ	Human:	Hepatocellular	[19]
		CD19^+^CD24^hi^CD38^hi^		
CD5	Activation of Stat3 via binding to IL-6	Human:	Lung, prostate	[57]
		CD19^+^CD5^+^IL-10+		
BTK	Repolarization of macrophages toward M2-type	Mouse:	Pancreatic (mice)	[82]
		CD19 + BTK+

### B cell effects on carcinogenesis

I) B cells are involved in de novo carcinogenesis in murine models of inflammation-associated squamous cell carcinoma

Several landmark studies also point to a role for B cells in facilitating carcinogenesis, mediated through effects on local inflammation [80, 81, 89, 90]. In the K14-HPV16 (HPV16) transgenic mouse model of inflammation-associated de novo epithelial carcinogenesis, the absence of T- and B-lymphocytes (generated by intercrossing with Rag1^−/−^ mice) limits immune cell infiltration and attenuates characteristic markers of premalignancy such as VEGF-A activity, matrix metalloproteinase activity, epithelial hyperproliferation, and development of angiogenic vasculature [89]. Subsequently, neoplastic progression is abrograted in HPV16/Rag1^−/−^ mice. Premalignant HPV16 skin was characterized by enhanced immunoglobulin (Ig) deposition, suggesting that B cells initiate Ig deposition into neoplastic tissue, paralleling inflammation and premalignant progression in HPV16 mice. Accordingly, skin from HPV16/Rag1^−/−^ mice had diminished Ig deposition and reduced immune cell infiltration. Adoptive transfer of B cells or serum, but not T cells from HPV16 mice into HPV16/Rag1^−/−^ mice restored Ig deposition, innate immune cell infiltration and premalignant characteristics and facilitated progression to epithelial carcinogenesis.

Based on these findings, *Affara* et al. [80] showed that B cell depletion with α-CD20 mAb restricted premalignant progression in K14-HPV16 transgenic mice. In contrast, α-CD20 mAb treatment did not affect growth of SCC tumors derived from K14-HPV16 mice that were orthotopically implanted into syngeneic WT mice. However, α-CD20 mAb treatment in combination with the chemotherapy (CTX) agents cisplatin, carboplatin, or paclitaxel, each ineffective on their own, resulted in tumor regression in mice orthotopically implanted with SCC tumors. Further analysis revealed that combinatorial α-CD20 mAb/CTX treatment was associated with increased tumor CD8^+^ infiltration compared to CTX treatment alone. Similar to aforementioned findings in a mouse model of prostate cancer [88], these results indicate the efficacy of augmenting chemotherapy with B-cell depletion strategies to improve outcomes.

Based on the findings implicating B cells in squamous carcinogenesis in mice [80, 89], and other previous studies showing that TNF-α deficient mice are resistant to chemical carcinogenesis of the skin [91], *Schioppa* et al. [90] investigators hypothesized that TNF-α might be involved in the tumor-promoting actions of B cells.

In a 7,12-dimethylbenz[α]anthracene/terephthalic acid (DMBA/TPA) mouse model of chemical-induced skin carcinogenesis, DMBA/TPA-induced papilloma development was blocked in B- and T-cell deficient Rag2^−/−^ mice. Reconstitution of DMBA/TPA-treated Rag2^−/−^ mice with B cells from DMBA/TPA-treated WT mice restored papilloma growth, whereas reconstitution with B cells from DMBA/TPA-treated TNFα^−/−^ mice failed to restore papilloma growth, thereby implicating TNFα-producing B cells as key mediators of tumorigenesis in this model. TNFα^−/−^ mice treated with DMBA/TPA also had reduced absolute numbers of splenic IL-10^+^CD19^+^ cells and increased absolute numbers of CD8^+^IFN-γ^+^ T cells in the spleen and lymph nodes compared to DMBA/TPA-treated WT mice. Together, these results suggest that TNFα secretion by B cells may regulate tumor growth through production of IL-10^+^ Bregs, which in turn attenuate CD8^+^IFN-γ^+^ T cell responses.

These studies suggest a unique B cell role in promoting a local inflammatory response, in turn leading to squamous carcinogenesis. Mechanisms underlying this role warrant further investigation but may involve enhanced inflammatory responses [81, 89] or local elaboration of TNF-α [90], both leading to enhanced myeloid cell infiltration and reduced CD8+ T cell infiltration. Interestingly, B cell depletion with α-CD20 mAb was efficacious in suppressing tumor growth as an adjunct to chemotherapy in the squamous carcinoma mouse model [80] and was efficacious as monotherapy in a mouse model of pancreatic cancer [92], however treatment with α-CD20 mAb accelerated tumor growth in mouse breast cancer models [64, 85]. Further investigations are warranted to determine how Breg induction may differ among tumor types and identify tumors that may be responsive to α-CD20 mAb treatment.

II) B cell infiltration of androgen-ablated prostate tumors in mice drives the development of castrate-resistant prostate cancer

In a mouse model of prostate cancer (myc-CaP), *Ammirante* et al. [93] demonstrated that castration of myc-CaP tumor-bearing mice results in the accumulation of an immune infiltrate comprised of B and T lymphocytes, NK cells, and myeloid cells in dying androgen-deprived primary tumors. This process precedes the emergence of castration-resistant prostate (CR-CaP) tumors. To further explore the role of tumor-infiltrating lymphocytes (TILs) in the emergence of CR-CaP, irradiated WT mice were reconstituted with bone marrow (BM) derived from either WT or Rag1^−/−^ mice and then inoculated with myc-CaP tumors, followed by castration 8 weeks later. Both groups showed identical growth of the primary tumor, however CR-CaP tumor growth after castration was significantly delayed in mice reconstituted with Rag1^−/−^ BM compared to WT BM. More rapid emergence of CR-CaP was restored when irradiated mice were reconstituted with TCRβ^−/−^ BM, however CR-CaP growth remained delayed when irradiated mice were reconstituted with J_H_^−/−^ BM, indicating that elimination of B cells rather than T cells was responsible for delayed growth of CR-CaP. Further mechanistic analysis revealed that secretion of lymphotoxin (LT) by infiltrating B cells stimulates LTβR on CaP cells to induce nuclear translocation of IKKα and activation of STAT3, thereby driving androgen-independent tumor growth after castration. Inhibition of IKKα, STAT3, LTβR, as well as B cell-specific LTβ ablation were each independently capable of delaying CR-CaP growth. This study invokes a completely novel mechanism by which local elaboration of a cytokine by infiltrating B cells may indirectly stimulate tumor growth. The relationship between androgen deprivation and B cell mobilization is poorly understood. Whether similar pathogenesis will be seen in human castration-resistant prostate cancer is not known.

III) B cell recruitment is regulated by HIF1α stabilization and promotes tumor growth in a murine model of pancreatic ductal adenocarcinoma

Hypoxia, a central feature of the tumor microenvironment, drives the expression of hypoxia-inducible factors on tumor cells including HIF1α, which regulates expression of genes involved in metabolism, angiogenesis, cell survival, and inflammation [94, 95]. *Lee* et al. [92] showed that hypoxia-driven HIF1α expression and stabilization occurred in the early growth stages of both murine Kras^G12D^ PDAC and human PDAC tumors, and that the deletion of pancreas-specific Hif1α in mice harboring Kras^G12D^ tumors resulted in accelerated tumor growth and was associated with an influx of intra-pancreatic B cells. Human PDAC tumors also showed significant intra-pancreatic B cell infiltration. B-cell depletion in Kras^G12D^/HIF1α-KO mice following treatment with α-CD20 mAb significantly decreased the number of grade 3 PanIN lesions and reduced the percentage of mice with microinvasive lesions, indicating that the accelerated tumor growth in HIF1α-KO mice is due at least in part related to increased intrapancreatic B-cell recruitment.

Increased secretion of the B-cell chemokine CXCL13 in HIF1α-KO mice was believed to be responsible for the prominent influx of intrapancreatic B cells, as CXCL13 levels by ELISA and immunohistochemical staining were significantly higher in Kras^G12D^/HIF1α-KO mice compared to Kras^G12D^ and WT mice. CXCL13 protein accumulation was also detected in the majority of human PDAC tumor samples.

Together, these observations indicate that hypoxia may accelerate the growth of HIF1α-deleted PDAC tumors in part by augmenting intrapancreatic B-cell accumulation, which appears to be driven by local expression of the B cell chemokine CXCL13. Therefore HIF1α expression and stabilization may actually inhibit tumor B cell infiltration and thus may be a protective mechanism in response to tissue hypoxia. The effects of HIF1α signaling on local B cell recruitment represent a unique and unanticipated effect of hypoxia on the composition of tumor immune infiltrates and subsequent anti-tumor response.

IV) IV. B cells are actively recruited to bladder tumors and promote tumorigenesis via secretion of IL-8

*Ou* et al. [22] demonstrated that human bladder cancer (BCa) tissues are capable of recruiting more B cells than surrounding normal bladder tissues. In turn, B cell infiltration in BCa tissues increases BCa cell invasiveness as demonstrated in in vitro chamber invasion assays using three separate human BCa cell lines cocultured with B cells. In addition, mice xenografted with human J82 BCa cells and co-implanted with B cells developed significantly more metastatic foci in vivo compared to mice implanted with J82 cells alone.

Increased expression of androgen receptor (AR) by tumor cells was associated with increased BCa invasiveness. AR signaling has been implicated in bladder carcinogenesis, as androgen deprivation has been shown to inhibit tumor growth in mouse models and xenografts [96].

Further investigation revealed that B cells augment AR expression in human BCa tissue by secreting IL-8, a pro-inflammatory chemokine shown to promote bladder carcinogenesis and metastasis [97, 98]. In turn, increased AR expression leads to upregulation of the matrix metalloproteinases MMP1 and MMP13, mediators of extracellular collagen degradation with known roles in tumor invasion, metastasis, and BCa progression [98, 99]. Knockdown of AR expression, inhibition of MMP1 and MMP13, or the use of anti-IL-8 neutralizing antibody all reversed the ability of B cells to increase BCa cell invasion.

This study highlighted a role for local elaboration of chemokines such as IL-8 by infiltrating B cells in promoting tumor invasion. It appears that the effect of IL-8 may be to upregulate AR expression on BCa cells and thereby increase expression of the AR-downstream pro-metastatic collagenases MMP1 and MMP13. The relationship between inflammation and upregulation of AR expression is unclear. Therapeutic strategies directed against Breg secretion of IL-8 and/or disruption of the IL-8/AR/MMP1/MMP13 pathway in BCa warrant further exploration.

### B regulatory cells in human solid tumor malignancies

I) CD20^±^ B cell and plasma cell infiltration of tumors have diverse effects on tumorigenesis

To date, most studies evaluating the prognostic significance of TILs have focused on T cells, while less attention has been devoted toward TIL-B cells. Using high resolution gene expression arrays, Schmidt et al. [11] showed that expression of a B cell “metagene” signature consisting of 60 genes in tumors from patients with node-negative breast cancer significantly correlated with metastasis-free survival. Tumor expression of the IgG^+^ plasma cell marker immunoglobulin kappa C (IGKC) provided prognostic information that was comparable with that of the B cell metagene signature in breast cancer patients. IGKC expression was also associated with better prognosis in non-small cell lung cancer (NSCLC) and colorectal adenocarcinoma (CRC) cohorts [11]. In another cohort of patients with CRC, higher density of tumor CD20^+^ B cell infiltration as measured by immunohistochemistry (IHC) correlated significantly with improved overall survival [7]. This data suggests that an immune response may actually be mediated by B cells in the aforementioned examples.

Increased tumor infiltration of CD20^+^ B cells has also been shown to portend a better prognosis among patients with epithelial ovarian cancer (EOC). Nielsen et al. [100] demonstrated that CD20^+^ B cells co-localized with CD8+ T cells in tumor specimens from patients with high-grade serous ovarian cancer (HGSC), raising the possibility that TIL-Bs act as antigen-presenting cells to facilitate the antitumor T cell cytolytic response. Consistent with the hypothesis that TIL-Bs may have an anti-tumor effect when co-localized with CD8^+^ T cells, Kroeger et al. [10] showed that TIL-B- and T-cells co-localize and arrange in tertiary lymphoid structures (TLS) surrounded by dense IgG^+^ plasma cell infiltrates in HGSC tumors. CD8^+^ TILs conferred a better prognosis only in the presence of adjacent CD4^+^ T cell TILs, CD20^+^ B cell TILs, and CD138^+^ plasma cells. Plasma cells adjacent to TLS may confer an anti-tumor effect via antibody-related mechanisms such as complement activation or antibody-dependent cellular cytotoxicity (ADCC) [10].

In contrast to the above observations, other studies have demonstrated an increase in CD20^+^ and CD138^+^ cell infiltration in association with a poorer prognosis. *Lundgren* et al. [8] demonstrated that in patients with EOC, increased tumoral CD20^+^ and CD138^+^ expression by IHC was associated with advanced tumor grade, and CD138 expression correlated significantly with reduced overall and cancer-specific survival. In patients with primary operable invasive ductal breast cancer, increased CD138^+^ B-cell infiltration was also independently associated with poorer recurrence-free survival [23]. In patients with prostate cancer, increased tumor CD20^+^ density correlated significantly with D’Amico high-risk categorization, and was predictive of treatment failure [24]. In addition, *Prueitt* et al. [12] showed that immunoglobin expression by TIL-B cells was increased in active smokers with PC, who are known to have a higher incidence of metastatic disease, compared to past or never smokers with PC.

Studies examining the prognostic significance and effects on anti-tumor immunity of tumor-infiltrating CD20^+^ B cells and CD138^+^ plasma cells have therefore yielded conflicting results. CD20^+^ B cells may function to present tumor antigen to cytotoxic T cells or other immune effector cells, and plasma cells may secrete antibodies that aid in the immune response against tumor cells [7, 10, 11, 100]. Alternatively, CD20^+^ and CD138^+^ TIL-Bs may function to suppress T cell anti-tumor responses as in Bregs or promote tumor progression by nurturing an inflammatory microenvironment. Better characterization of the immunophenotype of Breg subpopulations may clarify the nature of these conflicting results.

II) Bregs are upregulated in patients with solid tumors and are associated with more aggressive disease

In contrast to the conflicting results regarding significance of general tumor infiltration with CD20^+^ B cells and CD138^+^ plasma cells in humans, the majority of studies examining infiltration of tumors with B cells that have an established regulatory phenotype (CD38^hi^CD24^hi^, CD5 + CD1d^hi^, CD24^hi^CD27^+^, IL-10^+^) have demonstrated that Breg infiltration is associated with impaired anti-tumor immunity and more aggressive disease [13–22].

In patients with esophageal cancer, frequencies of peripheral blood IL-10^+^ Bregs were significantly greater compared with healthy controls [17]. IL-10^+^ Breg frequency also correlated with clinical staging and disease progression in esophageal cancer patients, as higher frequencies were seen in the peripheral blood of patients with Stage III/IV disease compared to early stage disease patients [17].

*Wei* et al. [16] demonstrated that in patients with ovarian cancer and ascites, CD19^+^IL-10^+^ Breg frequency was increased in ascitic fluid compared to peripheral blood. Furthermore, frequency of ascitic IL-10^+^ Bregs correlated with advanced stage and was associated with increased ascitic CD4^+^FoxP3^+^ Treg frequency and decreased ascitic CD8^+^IFN-γ^+^ T cell frequency. Ex vivo, ascitic fluid B cells from ovarian cancer patients suppressed IFN-γ production by CD8^+^ T cells.

Analysis of biopsy specimens from patients with tongue squamous cell carcinoma (TSCC) revealed increased frequency of CD19^+^IL-10^+^ Bregs in tumor tissue and metastatic lymph nodes compared to adjacent normal tissue [13]. Increased tumor IL-10^+^ Breg frequency was also associated with increased FoxP3^+^ Treg infiltration and reduced survival.

*Wang* et al. [20] showed that CD19^+^CD24^hi^CD38^hi^ IL-10^+^ Bregs were more prevalent in the peripheral blood of patients with gastric cancer versus healthy controls. In gastric cancer patients, IL-10^+^ Bregs were more prevalent in tumor tissues versus non-tumoral tissues and peripheral blood. Ex vivo, gastric cancer Bregs suppressed IFN-γ and TNF-α secretion by CD4^+^ cells and mediated conversion of CD4^+^ cells to CD4^+^FoxP3^+^ Tregs via TGF-β signaling.

In patients with CRC, CD24^hi^CD38^hi^ Bregs and CD24^hi^CD27^+^ Bregs were present in tumors and the frequency of CD24^hi^CD38^hi^ Bregs was significantly elevated in advanced stage tumors. Liver metastases from CRC had lower frequencies of B cells comprising the immune cell infiltrate compared to primary tumors, however the proportion of B cells with a regulatory phenotype was significantly increased in metastatic tissue, possibly indicating a shift in B cells toward a more immunosuppressive phenotype within the metastases [21].

Increased peripheral blood IL-10^+^ Bregs were observed in patients with NSCLC compared to healthy controls, and increased peripheral IL-10^+^ Bregs in NSCLC patients correlated with an increase in peripheral FoxP3^+^ Tregs and MDSCs [18]. Furthermore, increased peripheral IL-10^+^ Breg frequency was associated with more aggressive disease progression and advanced stage. While *Zhou* et al. [14] also identified increased frequency of peripheral Bregs in lung cancer patients versus controls, they unexpectedly also showed decreased frequency of peripheral Tregs, but speculated that lung tissues might show increased Tregs.

In tumor specimens from patients with hepatocellular cancer (HCC), the prevalence of intrahepatic B cells at the tumor margin was associated with tumor-invasive features [19]. Peripheral blood CD19^+^CD24^+^CD38^+^ Breg frequency was significantly greater in HCC patients versus healthy controls, and circulating Breg frequency correlated with advanced staging. Higher expression levels of CD40L were observed on Bregs versus non-Bregs as well.

SCID mice injected with human MHCC-97 L HCC cells mice together with human CD19^+^CD24^+^CD38^+^ Bregs demonstrated markedly larger tumor growth at 6 weeks and increased serum IL-10 levels compared to SCID mice injected with HCC cells and CD19^+^CD24^−^CD38^−^ non-Bregs. In in vitro co-culture studies, Bregs increased HCC cell proliferation, promoted secretion of IL-10 and TGF-β1 and decreased secretion of TNF-α compared to non-Bregs. CD40 was upregulated on HCC cells co-cultured with Bregs as well. Administration of anti-CD40L antibody blocked HCC tumor growth enhancement by Bregs in vivo, blocked enhancement of HCC cell proliferation by Bregs in vitro and decreased IL-10 and TGF-β1 secretion while promoting an increase of TNFα secretion. These results suggest that an expanded CD19^+^CD24^+^CD38^+^ peripheral blood Breg population in patients with HCC may migrate to the tumor margin and mediate tumor growth via local elaboration of immunosuppressive cytokines IL-10 and TGF-β, dependent on cognate interactions between CD40L and CD40 on Bregs and HCC cells respectively. Targeting of the CD40L/CD40 pathway may therefore be a possible therapeutic strategy in patients with advanced HCC.

Recently, a novel tumor-promoting PD-1^high^ Breg subset with CD5^high^CD24^−/+^CD27^high/+^CD38^dim^ phenotype was identified in tumor tissues of patients with HCC and correlated with advanced stage and early disease progression. Investigators identified increased tumor infiltration with PD-1^high^ Bregs which produced IL-10 upon interaction with PD-L1 or anti-PD1 antibody. Increased tumor infiltration with PD-1^high^ IL-10-producing Bregs was associated with reduced number and dysfunction of CD8^+^ cells. These findings identify a uniquely suppressive PD-1^+^ B cell subset in HCC pathogenesis. Therefore, PD-1 may also be viable target for the reduction of Breg activity in HCC [15].

These studies suggest that frequencies of B cells with regulatory phenotypes are increased in the peripheral blood of patients with various solid tumor types compared to age-matched controls. Also, there is evidence that B cells with regulatory phenotypes accumulate in the tumor tissues and peri-tumoral environment. It is not clear if Bregs are actively promoting tumor growth in humans or if an increase in Bregs is merely an immune response against the tumor, however aforementioned ex vivo and xenograft assays demonstrating suppressive properties of Bregs gives credence to the former hypothesis.

## Conclusions

It has become clear that in both mouse models and in humans that B cells can mediate immunosuppression through modulation of innate and/or adaptive immune responses in support of tumor growth. In several murine model systems B cells are actively recruited to tumors and directly acquire suppressive activity within the tumor bed [69]. Signaling through diverse pathways such as BTK, NF-kB and/or STAT3 has been implicated in the generation of the Breg phenotype.

A variety of cytokines secreted by Bregs have been implicated in the suppression of anti-tumor immunity including IL-10, IL-35, IL-6, and TGF-β. Additionally, Bregs may express a variety of suppressive ligands including PD-L1, PD-1, CD80, CD86, LAP-TGF-β, Fas-L, CD40L, and OX40L. Bregs may also express proteases such as Granzyme-B that directly impair T cell function [62].

Bregs may support expansion of suppressive Tregs and MDSCs, suppress stimulatory Th_1_/Th_17_ cells, promote skewing of T-helper cells and macrophages toward suppressive Th_2_/M2 types, and/or may interact directly with effector CD4^+^ and CD8^+^ T cell and/or NK cells to suppress anti-tumor immunity.

B cells may also directly promote carcinogenesis through local elaboration of inflammatory mediators such as TNF-α in squamous cell skin cancer [90], lymphotoxin in prostate cancer [93], and IL-8 in bladder cancer [22]. Antibody production and subsequent deposition of immune-complexes in tumor tissue is another mechanism whereby B cells may promote inflammation and neoplastic progression. B cells may also facilitate tumorigenesis through the upregulation of pro-angiogenic genes.

The list of human tumors infiltrated with B cells is rapidly expanding. B cell infiltration of tumors has been associated with improved prognosis or alternatively with enhanced tumor aggressiveness in different studies. Studies examining B cells with regulatory phenotypes however suggest uniformly that Breg infiltration may enhance tumor progression.

B cell depletion can be accomplished using α-CD20 mAb such as rituximab and obinatuzumab, or through use of inhibitors of signal transduction such as ibrutinib.

B cell depletion strategies deployed in combination with current immune therapies requires further study. Not all forms of B cell depletion may be equally effective, as demonstrated by the failure or limited effectiveness of α-CD20 mAb therapy in several tumor models. Further investigations are necessary to identify Breg-specific targets that may be selectively used to deplete key B cell subpopulations with regulatory function, and/or to modulate B cell-T effector cell cross-talk through co-stimulatory ligands, cytokines, and/or chemokines to augment anti-tumor effector responses.

## Abbreviations

ADCC, antibody dependent cellular cytotoxicity; AR, androgen receptor; B10 B cells, IL-10 producing B cells; BCa, bladder cancer; BCDM, B cell deficient mice; Bregs, B regulatory cells; BTK, Bruton’s tyrosine kinase; CIA, collagen induced arthritis; CLL, chronic lymphocytic leukemia; CRC, colorectal adenocarcinoma; CTL, cytotoxic T lymphocytes; DLBCL, diffuse large B cell lymphoma; EAE, experimental autoimmune disease; Fas-L, Fas ligand; GrB+, Granzyme-B producing Breg cells; HNSCC, head and neck squamous cell carcinoma; IBD, inflammatory bowel disease; IGKC, immunoglobulin kappa C; LLC, Lewis lung cancer; LT, lymphotoxin; MC, Myc-Cap; MDSC, myeloid-derived suppressor cells; NK cells, nature killer cells; NSCLC, non-small cell lung cancer; PanIN, pancreatic intraepithelial neoplasia; PC, prostate carcinoma; PDAC, pancreatic ductal adenocarcinoma; RSV, resveratrol; SCC, squamous cell carcinoma; SLE, systemic lupus erythematosus; TAA, tumor associated antigen; TAM, tumor associated macrophages; tBregs, tumor-evoked Breg cells; TDLN, tumor draining lymph nodes; TIL, tumor infiltrating lymphocytes; TIL-B, tumor infiltrating B lymphocytes; Tregs, T regulatory cells; WT, wild type; α-CD20 mAb, anti-CD20 monoclonal antibody
